# (*E*)-(2,5-Difluoro­benz­yl)[(2-eth­oxy­naphthalen-1-yl)methyl­idene]amine

**DOI:** 10.1107/S1600536813001967

**Published:** 2013-02-09

**Authors:** Merve Pekdemir, Şamil Işık, Mustafa Macit, Ayşen Alaman Ağar, Mustafa Serkan Soylu

**Affiliations:** a Department of Physics, Faculty of Arts and Sciences, Ondokuz Mayıs University, Kurupelit, TR-55139 Samsun, Turkey; b Department of Chemistry, Art and Science Faculty, Ondokuz Mayıs University, Kurupelit, TR-55139 Samsun, Turkey; c Giresun University, Arts and Science Faculty, Department of Physics, Giresun, Turkey

## Abstract

In the title mol­ecule, C_20_H_17_F_2_NO, which adopts an *E* conformation with respect to the imine C=N double bond, the mean planes of the naphthalene ring system and the difluoro­phenyl ring form a dihedral angle of 85.82 (7)°. An intra­molecular C—H⋯N hydrogen bond occurs. In the crystal, weak C—H⋯F hydrogen bonds link the mol­ecules into zigzag chains along [010].

## Related literature

For structural studies of Schiff bases by our group, see: Gül *et al.* (2007 ▶); Kantar *et al.* (2012 ▶); Kargılı *et al.* (2012 ▶); Pekdemir *et al.* (2012 ▶); Vesek *et al.* (2012 ▶). For classification of hydrogen-bonding patterns, see: Bernstein *et al.* (1995 ▶). 
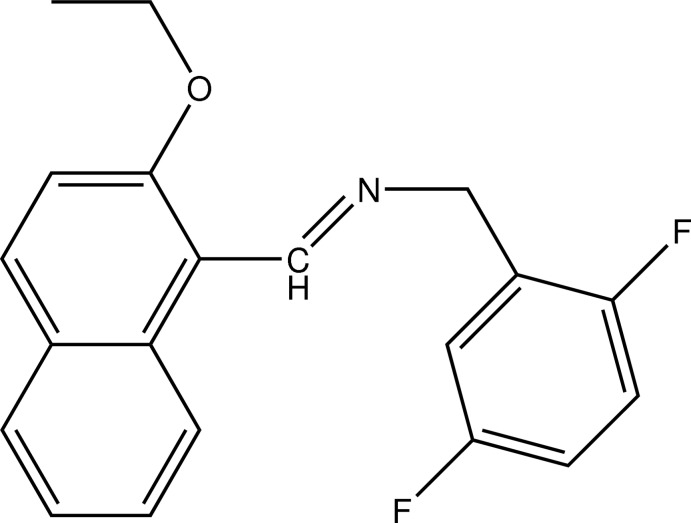


## Experimental

### 

#### Crystal data

C_20_H_17_F_2_NO
*M* *_r_* = 325.35
Monoclinic, 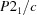

*a* = 12.5963 (8) Å
*b* = 14.3010 (8) Å
*c* = 9.8693 (8) Å
β = 108.672 (8)°
*V* = 1684.3 (2) Å^3^
*Z* = 4
Mo *K*α radiation
μ = 0.09 mm^−1^
*T* = 293 K
0.30 × 0.25 × 0.25 mm

#### Data collection

Oxford Diffraction SuperNova Eos diffractometer
Absorption correction: multi-scan (*CrysAlis PRO*; Oxford Diffraction, 2007 ▶) *T*_min_ = 0.703, *T*_max_ = 1.000
5942 measured reflections
2958 independent reflections
1997 reflections with *I* > 2σ(*I*)
*R*_int_ = 0.018

#### Refinement

*R*[*F* ^2^ > 2σ(*F* ^2^)] = 0.058
*wR*(*F* ^2^) = 0.151
*S* = 1.06
2958 reflections
217 parameters
H-atom parameters constrained
Δρ_max_ = 0.18 e Å^−3^
Δρ_min_ = −0.18 e Å^−3^

### 

Data collection: *CrysAlis PRO* (Oxford Diffraction, 2007 ▶); cell refinement: *CrysAlis PRO*; data reduction: *CrysAlis PRO*; program(s) used to solve structure: *SHELXS97* (Sheldrick, 2008 ▶); program(s) used to refine structure: *SHELXL97* (Sheldrick, 2008 ▶); molecular graphics: *ORTEP-3 for Windows* (Farrugia, 2012 ▶); software used to prepare material for publication: *WinGX* (Farrugia, 2012 ▶).

## Supplementary Material

Crystal structure: contains datablock(s) I, global. DOI: 10.1107/S1600536813001967/cv5376sup1.cif

Structure factors: contains datablock(s) I. DOI: 10.1107/S1600536813001967/cv5376Isup2.hkl

Supplementary material file. DOI: 10.1107/S1600536813001967/cv5376Isup3.cml

Additional supplementary materials:  crystallographic information; 3D view; checkCIF report

## Figures and Tables

**Table 1:** Hydrogen-bond geometry (Å, °)

*D*—H⋯*A*	*D*—H	H⋯*A*	*D*⋯*A*	*D*—H⋯*A*
C3—H3⋯N1	0.93	2.32	2.955 (3)	125
C6—H6⋯F1^i^	0.93	2.61	3.505 (4)	162

Symmetry code: (i) 
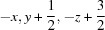
.

**Fig. 1.**
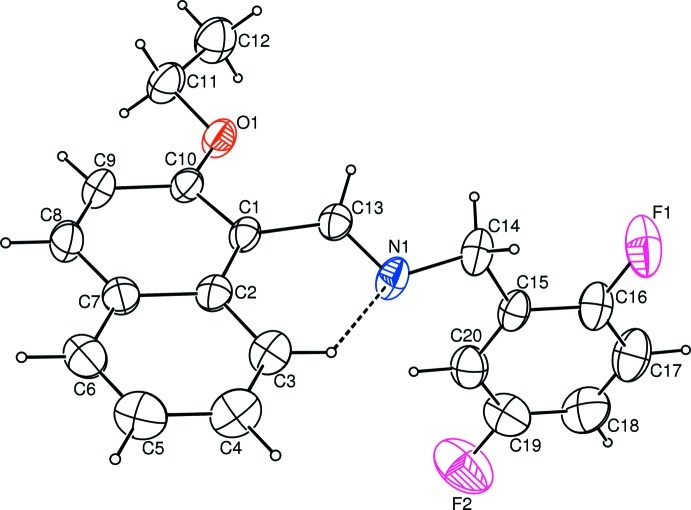
The molecular structure of (I), showing the atom-numbering scheme. Displacement ellipsoids are drawn at the 50% probability. Dashed line denotes hydrogen bond.

**Fig. 2.**
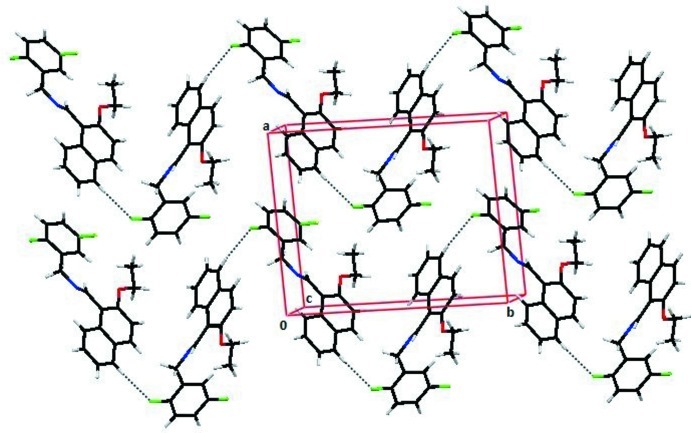
A portion of the crystal packing showing weak intermolecular C—H···F hydrogen bonds as dashed lines.

## supplementary crystallographic information

### Comment

In continuation of our structural study of Schiff bases (Gül *et al.*, 2007; Kantar *et al.*, 2012; Pekdemir *et al.*, 2012; Vesek *et al.*, 2012), herewith we present the title compound, (I).

In (I) (Fig. 1), the C13═N1 bond distance of 1.262 (3) Å is shorter than the standart 1.28 Å value of C═N double bond, similar to the corresponding bond distance in (E)—N-[(2- ethoxynaphthalen-1-yl)methylidene]-2-ethylaniline [1.261 (3) Å; Kargılı *et al.*,2012]. The dihedral angle between the naphthalene ring system and the benzene ring is 85.82 (7)°. The C13—N1—C14—C15 torsion angle is 179.4 (3)°. The molecular conformation is supported by an intramolecular C—H···N hydrogen bond, which generates S(6) ring (Bernstein *et al.*, 1995).

In the crystal, weak intermolecular C—H···F hydrogen bonds (Table 1) link the molecules into zigzag chains in [010] (Fig. 2).

### Experimental

(*E*)-1-(2,5-difluorophenyl)-*N*-((2-ethoxynaphthalen-1-yl) methylene)methanamine was prepared by refluxing a mixture of a solution containing 2-ethoxy-1-naphthaldehyde (20.0 mg, 0.1 mmol) in ethanol (20 ml) and a solution containing 2,5-difluorobenzylamine (14.3 mg, 0.1 mmol) in ethanol (20 ml). The reaction mixture was stirred for 5 h under reflux. Single crystals of the title compound for X-ray analysis were obtained by slow evaporation of an ethanol solution (Yield 74%; m.p.356 - 358 K).

### Refinement

All H atoms were placed in calculated positions with C—H = 0.93–0.97 Å, and constrained to ride on their parents atoms, with *U*_iso_(H) = 1.2 – 1.5*U*_eq_(C).

### Crystal data

**Table:**

C_20_H_17_F_2_NO	*F*(000) = 680
*M**_r_* = 325.35	*D*_x_ = 1.283 Mg m^−^^3^
Monoclinic, *P*2_1_/*c*	Mo *K*α radiation, λ = 0.71073 Å
Hall symbol: -P 2ybc	Cell parameters from 1869 reflections
*a* = 12.5963 (8) Å	θ = 3.3–29.3°
*b* = 14.3010 (8) Å	µ = 0.09 mm^−^^1^
*c* = 9.8693 (8) Å	*T* = 293 K
β = 108.672 (8)°	Block, yellow
*V* = 1684.3 (2) Å^3^	0.30 × 0.25 × 0.25 mm
*Z* = 4	

### Data collection

**Table:**

Oxford Diffraction SuperNova Eos diffractometer	2958 independent reflections
Radiation source: fine-focus sealed tube	1997 reflections with *I* > 2σ(*I*)
Graphite monochromator	*R*_int_ = 0.018
Detector resolution: 16.0454 pixels mm^-1^	θ_max_ = 25.0°, θ_min_ = 3.3°
ω scans	*h* = −13→14
Absorption correction: multi-scan (*CrysAlis PRO*; Oxford Diffraction, 2007)	*k* = −17→17
*T*_min_ = 0.703, *T*_max_ = 1.000	*l* = −11→11
5942 measured reflections	

### Refinement

**Table:**

Refinement on *F*^2^	Primary atom site location: structure-invariant direct methods
Least-squares matrix: full	Secondary atom site location: difference Fourier map
*R*[*F*^2^ > 2σ(*F*^2^)] = 0.058	Hydrogen site location: inferred from neighbouring sites
*wR*(*F*^2^) = 0.151	H-atom parameters constrained
*S* = 1.06	*w* = 1/[σ^2^(*F*_o_^2^) + (0.0491*P*)^2^ + 0.5882*P*] where *P* = (*F*_o_^2^ + 2*F*_c_^2^)/3
2958 reflections	(Δ/σ)_max_ < 0.001
217 parameters	Δρ_max_ = 0.18 e Å^−^^3^
0 restraints	Δρ_min_ = −0.18 e Å^−^^3^

### Special details

**Table:**

Geometry. All e.s.d.'s (except the e.s.d. in the dihedral angle between two l.s. planes) are estimated using the full covariance matrix. The cell e.s.d.'s are taken into account individually in the estimation of e.s.d.'s in distances, angles and torsion angles; correlations between e.s.d.'s in cell parameters are only used when they are defined by crystal symmetry. An approximate (isotropic) treatment of cell e.s.d.'s is used for estimating e.s.d.'s involving l.s. planes.
Refinement. Refinement of *F*^2^ against ALL reflections. The weighted *R*-factor *wR* and goodness of fit *S* are based on *F*^2^, conventional *R*-factors *R* are based on *F*, with *F* set to zero for negative *F*^2^. The threshold expression of *F*^2^ > σ(*F*^2^) is used only for calculating *R*-factors(gt) *etc*. and is not relevant to the choice of reflections for refinement. *R*-factors based on *F*^2^ are statistically about twice as large as those based on *F*, and *R*- factors based on ALL data will be even larger.

### Fractional atomic coordinates and isotropic or equivalent isotropic displacement parameters (Å^2^)

**Table:**

	*x*	*y*	*z*	*U*_iso_*/*U*_eq_	
C1	0.05443 (18)	0.09513 (16)	0.9269 (2)	0.0475 (6)	
C2	−0.04096 (19)	0.08982 (16)	0.8003 (3)	0.0505 (6)	
C3	−0.0486 (2)	0.02790 (19)	0.6849 (3)	0.0648 (7)	
H3	0.0107	−0.0121	0.6900	0.078*	
C4	−0.1420 (3)	0.0264 (2)	0.5666 (3)	0.0792 (9)	
H4	−0.1445	−0.0140	0.4918	0.095*	
C5	−0.2335 (2)	0.0840 (2)	0.5559 (3)	0.0802 (9)	
H5	−0.2967	0.0816	0.4750	0.096*	
C6	−0.2300 (2)	0.1434 (2)	0.6633 (3)	0.0690 (8)	
H6	−0.2917	0.1812	0.6561	0.083*	
C7	−0.1346 (2)	0.14949 (18)	0.7868 (3)	0.0558 (6)	
C8	−0.1289 (2)	0.2129 (2)	0.8967 (3)	0.0669 (7)	
H8	−0.1897	0.2520	0.8879	0.080*	
C9	−0.0383 (2)	0.21936 (19)	1.0152 (3)	0.0657 (7)	
H9	−0.0369	0.2628	1.0859	0.079*	
C10	0.0540 (2)	0.15986 (17)	1.0311 (3)	0.0529 (6)	
C11	0.1491 (2)	0.2230 (2)	1.2652 (3)	0.0759 (8)	
H11A	0.0884	0.2049	1.3004	0.091*	
H11B	0.1386	0.2879	1.2353	0.091*	
C12	0.2588 (3)	0.2110 (2)	1.3797 (3)	0.0938 (10)	
H12A	0.2606	0.2491	1.4604	0.141*	
H12B	0.3182	0.2296	1.3441	0.141*	
H12C	0.2685	0.1466	1.4084	0.141*	
C13	0.15149 (19)	0.03193 (16)	0.9571 (3)	0.0515 (6)	
H13	0.1901	0.0185	1.0524	0.062*	
C14	0.2785 (2)	−0.07076 (19)	0.9136 (3)	0.0690 (8)	
H14A	0.2510	−0.1338	0.8875	0.083*	
H14B	0.3083	−0.0676	1.0171	0.083*	
C15	0.37095 (19)	−0.05036 (18)	0.8517 (3)	0.0578 (7)	
C16	0.4542 (2)	−0.1146 (2)	0.8634 (3)	0.0749 (8)	
C17	0.5395 (3)	−0.1034 (3)	0.8079 (4)	0.0963 (11)	
H17	0.5931	−0.1499	0.8179	0.116*	
C18	0.5443 (3)	−0.0221 (3)	0.7371 (4)	0.1016 (12)	
H18	0.6008	−0.0124	0.6967	0.122*	
C19	0.4647 (3)	0.0442 (3)	0.7271 (4)	0.0894 (10)	
C20	0.3785 (2)	0.0321 (2)	0.7833 (3)	0.0738 (8)	
H20	0.3259	0.0792	0.7750	0.089*	
F1	0.45086 (15)	−0.19516 (13)	0.9363 (3)	0.1126 (7)	
F2	0.46988 (19)	0.12570 (19)	0.6602 (3)	0.1524 (10)	
N1	0.18605 (17)	−0.00533 (16)	0.8631 (2)	0.0647 (6)	
O1	0.14961 (14)	0.16450 (12)	1.14686 (18)	0.0653 (5)	

### Atomic displacement parameters (Å^2^)

**Table:**

	*U*^11^	*U*^22^	*U*^33^	*U*^12^	*U*^13^	*U*^23^
C1	0.0479 (13)	0.0437 (13)	0.0606 (14)	0.0002 (11)	0.0309 (11)	0.0012 (11)
C2	0.0504 (13)	0.0479 (13)	0.0615 (14)	−0.0059 (11)	0.0294 (11)	0.0034 (12)
C3	0.0580 (15)	0.0659 (17)	0.0731 (17)	−0.0086 (13)	0.0248 (13)	−0.0090 (14)
C4	0.0711 (19)	0.086 (2)	0.079 (2)	−0.0214 (17)	0.0224 (16)	−0.0187 (17)
C5	0.0602 (18)	0.094 (2)	0.080 (2)	−0.0175 (17)	0.0126 (15)	0.0027 (19)
C6	0.0533 (15)	0.0746 (18)	0.0803 (19)	−0.0004 (14)	0.0233 (14)	0.0203 (17)
C7	0.0538 (14)	0.0550 (15)	0.0662 (15)	0.0011 (12)	0.0299 (12)	0.0122 (13)
C8	0.0624 (16)	0.0677 (18)	0.0804 (19)	0.0171 (14)	0.0367 (15)	0.0094 (15)
C9	0.0726 (18)	0.0666 (17)	0.0673 (17)	0.0152 (15)	0.0356 (15)	−0.0071 (14)
C10	0.0554 (14)	0.0545 (15)	0.0566 (14)	0.0043 (12)	0.0289 (12)	0.0005 (12)
C11	0.083 (2)	0.080 (2)	0.0684 (18)	0.0049 (16)	0.0296 (15)	−0.0219 (16)
C12	0.091 (2)	0.105 (3)	0.079 (2)	0.006 (2)	0.0167 (17)	−0.0266 (19)
C13	0.0515 (13)	0.0468 (14)	0.0621 (14)	−0.0025 (11)	0.0265 (11)	−0.0008 (12)
C14	0.0623 (16)	0.0550 (16)	0.098 (2)	0.0051 (13)	0.0371 (15)	−0.0117 (15)
C15	0.0460 (13)	0.0582 (15)	0.0671 (16)	−0.0007 (12)	0.0152 (11)	−0.0197 (13)
C16	0.0596 (17)	0.0640 (18)	0.106 (2)	0.0023 (15)	0.0332 (16)	−0.0129 (17)
C17	0.0589 (19)	0.105 (3)	0.135 (3)	0.0102 (19)	0.044 (2)	−0.017 (2)
C18	0.062 (2)	0.142 (4)	0.112 (3)	−0.005 (2)	0.0430 (19)	−0.001 (3)
C19	0.067 (2)	0.103 (3)	0.096 (2)	−0.0109 (19)	0.0223 (17)	0.017 (2)
C20	0.0589 (16)	0.076 (2)	0.0832 (19)	0.0036 (15)	0.0179 (15)	−0.0035 (16)
F1	0.0883 (13)	0.0712 (12)	0.192 (2)	0.0199 (10)	0.0639 (14)	0.0089 (13)
F2	0.1138 (17)	0.161 (2)	0.186 (2)	−0.0072 (16)	0.0539 (17)	0.078 (2)
N1	0.0534 (12)	0.0723 (15)	0.0744 (14)	0.0099 (11)	0.0288 (11)	−0.0132 (12)
O1	0.0654 (11)	0.0716 (12)	0.0626 (11)	0.0083 (9)	0.0257 (9)	−0.0139 (9)

### Geometric parameters (Å, º)

**Table:**

C1—C10	1.385 (3)	C11—H11B	0.9700
C1—C2	1.432 (3)	C12—H12A	0.9600
C1—C13	1.472 (3)	C12—H12B	0.9600
C2—C3	1.421 (3)	C12—H12C	0.9600
C2—C7	1.427 (3)	C13—N1	1.262 (3)
C3—C4	1.366 (4)	C13—H13	0.9300
C3—H3	0.9300	C14—N1	1.452 (3)
C4—C5	1.394 (4)	C14—C15	1.507 (3)
C4—H4	0.9300	C14—H14A	0.9700
C5—C6	1.348 (4)	C14—H14B	0.9700
C5—H5	0.9300	C15—C16	1.371 (3)
C6—C7	1.414 (3)	C15—C20	1.377 (4)
C6—H6	0.9300	C16—C17	1.363 (4)
C7—C8	1.398 (4)	C16—F1	1.366 (3)
C8—C9	1.351 (4)	C17—C18	1.368 (5)
C8—H8	0.9300	C17—H17	0.9300
C9—C10	1.408 (3)	C18—C19	1.361 (5)
C9—H9	0.9300	C18—H18	0.9300
C10—O1	1.371 (3)	C19—F2	1.352 (4)
C11—O1	1.438 (3)	C19—C20	1.379 (4)
C11—C12	1.489 (4)	C20—H20	0.9300
C11—H11A	0.9700		
			
C10—C1—C2	118.8 (2)	C11—C12—H12A	109.5
C10—C1—C13	117.4 (2)	C11—C12—H12B	109.5
C2—C1—C13	123.7 (2)	H12A—C12—H12B	109.5
C3—C2—C7	117.1 (2)	C11—C12—H12C	109.5
C3—C2—C1	123.7 (2)	H12A—C12—H12C	109.5
C7—C2—C1	119.2 (2)	H12B—C12—H12C	109.5
C4—C3—C2	120.9 (3)	N1—C13—C1	124.7 (2)
C4—C3—H3	119.5	N1—C13—H13	117.6
C2—C3—H3	119.5	C1—C13—H13	117.6
C3—C4—C5	121.3 (3)	N1—C14—C15	112.0 (2)
C3—C4—H4	119.3	N1—C14—H14A	109.2
C5—C4—H4	119.3	C15—C14—H14A	109.2
C6—C5—C4	119.7 (3)	N1—C14—H14B	109.2
C6—C5—H5	120.2	C15—C14—H14B	109.2
C4—C5—H5	120.2	H14A—C14—H14B	107.9
C5—C6—C7	121.4 (3)	C16—C15—C20	116.4 (3)
C5—C6—H6	119.3	C16—C15—C14	120.4 (3)
C7—C6—H6	119.3	C20—C15—C14	123.3 (2)
C8—C7—C6	121.8 (2)	C17—C16—F1	118.2 (3)
C8—C7—C2	118.7 (2)	C17—C16—C15	124.6 (3)
C6—C7—C2	119.6 (2)	F1—C16—C15	117.3 (3)
C9—C8—C7	122.4 (2)	C16—C17—C18	118.4 (3)
C9—C8—H8	118.8	C16—C17—H17	120.8
C7—C8—H8	118.8	C18—C17—H17	120.8
C8—C9—C10	119.6 (2)	C19—C18—C17	118.5 (3)
C8—C9—H9	120.2	C19—C18—H18	120.8
C10—C9—H9	120.2	C17—C18—H18	120.8
O1—C10—C1	116.1 (2)	F2—C19—C18	119.1 (3)
O1—C10—C9	122.5 (2)	F2—C19—C20	118.2 (3)
C1—C10—C9	121.4 (2)	C18—C19—C20	122.7 (3)
O1—C11—C12	107.7 (2)	C15—C20—C19	119.5 (3)
O1—C11—H11A	110.2	C15—C20—H20	120.3
C12—C11—H11A	110.2	C19—C20—H20	120.3
O1—C11—H11B	110.2	C13—N1—C14	116.6 (2)
C12—C11—H11B	110.2	C10—O1—C11	118.26 (19)
H11A—C11—H11B	108.5		
			
C10—C1—C2—C3	178.3 (2)	C8—C9—C10—C1	0.6 (4)
C13—C1—C2—C3	−5.1 (3)	C10—C1—C13—N1	−153.0 (2)
C10—C1—C2—C7	−1.6 (3)	C2—C1—C13—N1	30.4 (3)
C13—C1—C2—C7	175.0 (2)	N1—C14—C15—C16	−166.9 (2)
C7—C2—C3—C4	0.1 (4)	N1—C14—C15—C20	14.0 (4)
C1—C2—C3—C4	−179.8 (2)	C20—C15—C16—C17	−2.6 (4)
C2—C3—C4—C5	−1.1 (4)	C14—C15—C16—C17	178.3 (3)
C3—C4—C5—C6	0.7 (5)	C20—C15—C16—F1	177.2 (2)
C4—C5—C6—C7	0.8 (4)	C14—C15—C16—F1	−1.9 (4)
C5—C6—C7—C8	178.0 (3)	F1—C16—C17—C18	−178.8 (3)
C5—C6—C7—C2	−1.8 (4)	C15—C16—C17—C18	1.0 (5)
C3—C2—C7—C8	−178.5 (2)	C16—C17—C18—C19	0.9 (5)
C1—C2—C7—C8	1.4 (3)	C17—C18—C19—F2	178.6 (3)
C3—C2—C7—C6	1.3 (3)	C17—C18—C19—C20	−1.1 (5)
C1—C2—C7—C6	−178.8 (2)	C16—C15—C20—C19	2.2 (4)
C6—C7—C8—C9	179.9 (3)	C14—C15—C20—C19	−178.7 (3)
C2—C7—C8—C9	−0.3 (4)	F2—C19—C20—C15	179.8 (3)
C7—C8—C9—C10	−0.8 (4)	C18—C19—C20—C15	−0.5 (5)
C2—C1—C10—O1	−177.18 (19)	C1—C13—N1—C14	−175.9 (2)
C13—C1—C10—O1	6.1 (3)	C15—C14—N1—C13	−130.8 (2)
C2—C1—C10—C9	0.6 (3)	C1—C10—O1—C11	−173.3 (2)
C13—C1—C10—C9	−176.2 (2)	C9—C10—O1—C11	9.0 (3)
C8—C9—C10—O1	178.2 (2)	C12—C11—O1—C10	176.3 (2)

### Hydrogen-bond geometry (Å, º)

**Table:**

*D*—H···*A*	*D*—H	H···*A*	*D*···*A*	*D*—H···*A*
C3—H3···N1	0.93	2.32	2.955 (3)	125
C6—H6···F1^i^	0.93	2.61	3.505 (4)	162

Symmetry code: (i) −*x*, *y*+1/2, −*z*+3/2.

## References

[bb1] Bernstein, J., Davis, R. E., Shimoni, L. & Chang, N.-L. (1995). *Angew. Chem. Int. Ed. Engl.* **34**, 1555–1573.

[bb2] Farrugia, L. J. (2012). *J. Appl. Cryst.* **45**, 849–854.

[bb3] Gül, Z. S., Erşahin, F., Ağar, E. & Işık, Ş. (2007). *Acta Cryst.* E**63**, o2902.

[bb4] Kantar, E. N., Köysal, Y., Gümüş, S., Ağar, E. & Soylu, M. S. (2012). *Acta Cryst.* E**68**, o1587. 10.1107/S160053681201882X PMC3379200 22719398

[bb5] Kargılı, H., Macit, M., Alpaslan, G., Kazak, C. & Erdönmez, A. (2012). *Acta Cryst.* E**68**, o3176. 10.1107/S1600536812043097 PMC3515269 23284489

[bb6] Oxford Diffraction (2007). *CrysAlis PRO* Oxford Diffraction Ltd, Abingdon, England.

[bb7] Pekdemir, M., Işık, Ş. & Alaman Ağar, A. (2012). *Acta Cryst.* E**68**, o2148. 10.1107/S1600536812026876 PMC3393956 22798821

[bb8] Sheldrick, G. M. (2008). *Acta Cryst.* A**64**, 112–122. 10.1107/S0108767307043930 18156677

[bb9] Vesek, H., Kazak, C., Alaman Ağar, A., Macit, M. & Soylu, M. S. (2012). *Acta Cryst.* E**68**, o2518. 10.1107/S1600536812032114 PMC3414968 22904955

